# Nuclear functions regulated by the VRK1 kinase

**DOI:** 10.1080/19491034.2024.2353249

**Published:** 2024-05-16

**Authors:** Pedro A. Lazo

**Affiliations:** a Instituto de Biología Molecular y Celular del Cáncer, Consejo Superior de Investigaciones Científicas (CSIC) - Universidad de Salamanca, Salamanca, Spain; b Instituto de Investigación Biomédica de Salamanca (IBSAL), Hospital Universitario de Salamanca, Salamanca, Spain

**Keywords:** Cajal bodies, cell cycle, chromatin, DNA damage, histones, VRK1

## Abstract

In the nucleus, the VRK1 Ser-Thr kinase is distributed in nucleoplasm and chromatin, where it has different roles. VRK1 expression increases in response to mitogenic signals. VRK1 regulates cyclin D1 expression at G0 exit and facilitates chromosome condensation at the end of G2 and G2/M progression to mitosis. These effects are mediated by the phosphorylation of histone H3 at Thr3 by VRK1, and later in mitosis by haspin. VRK1 regulates the apigenetic patterns of histones in processes requiring chromating remodeling, such as transcription, replication and DNA repair. VRK1 is overexpressed in tumors, facilitating tumor progression and resistance to genotoxic treatments. VRK1 also regulates the organization of Cajal bodies assembled on coilin, which are necessary for the assembly of different types of RNP complexes. VRK1 pathogenic variants cuase defects in Cajal bodies, functionally altering neurons with long axons and leading to neurological diseases, such as amyotrophic laterla sclerosis, spinal muscular atrophy, distal hereditay motor neuropathies and Charcot-Marie-Tooth.

## Introduction

Dynamic remodeling of chromatin is required for its adaptation to various normal and pathological functions. These functions include gene transcription and silencing, replication, recombination, and DNA damage responses, for which there are different types of DNA repair pathways. Chromatin is organized by DNA-wrapping nucleosomes formed by histones, which can be modulated by several types of posttranslational modifications. These modifications permit multiple locally different conformations to adapt to specific functions regulated by different epigenetic enzyme families. These chromatin remodeling processes require functional and temporal coordination. VRK1 (vaccinia-related kinase 1), also known in *Drosophila melanogaster* as NHK1 (nucleosomal histone kinase 1) [1]. In pluricellular eukaryotes, this Ser-Thr kinase family appeared later in evolution, constituting an isolated branch that diverged from casein kinases, after which, each evolved independently [2]. The VRK protein family is composed of three members, but VRK2 and VRK3 are mostly cytosolic [3]. Nuclear VRK1 regulates the dynamic reorganization of chromatin required for several types of functions, either physiological such as transcription, replication and recombination, or pathological, such as the response to different types of DNA damage [4,5], as well as to other functions such as chromatin interaction with components of the nuclear envelope [6,7], and Cajal bodies [8,9].

## VRK1, chromatin and epigenetic modifications of histones

Specific patterns of histone epigenetic modifications are associated with different biological functions [10,11], including transcription, replication, recombination, and DNA damage responses. Alterations of these patterns can lead to pathological situations such as cancer [12–14] and neurological diseases [15]. Chromatin organization, in which nucleosomes are the basic unit, underlies all biological processes implicated in its functions and requires a complex coordination in which kinases play a relevant role [11,16,17]. VRK1 is a Ser-Thr kinase, mostly located in the nucleus and chromatin, where it plays different roles, but there is also a cytosolic subpopulation at lower concentrations [4,5]. VRK1 [18] and NHK-1 [19] facilitate chromosome condensation in human and *Drosophila melanogaster* cells, respectively. The VRK1 protein has a low-complexity C-terminal flexible region that folds over the catalytic site, and has several alternative conformations permitting different protein interactions, which are critical for the structural stability, regulation of the kinase activity [20], and selection of interacting proteins [4]. At the distal end of the VRK1 C-terminal flexible region, there is a basic Arg-rich motif that directly interacts with the nucleosome acidic patch [21]. In this context, VRK1 directly interacts with histones H2A [22], H3 [23,24], H4 [25–27] and H2AX [28]. VRK1 directly interacts with histones H2A and H3, and phosphorylates histone H2A in Thr120 [22] and H3 in Thr3 [24,28], two modifications associated with local chromatin remodeling. VRK1 also phosphorylates histone H2AX at Ser139 (γH2AX) in the response to DNA damage [28], which is considered the guardian of the genome [29]. In centromeres, defects in H2A-Thr120 phosphorylation by BUB1 alter chromosome segregation, generating multinucleated cells [30].

**Figure 1.**
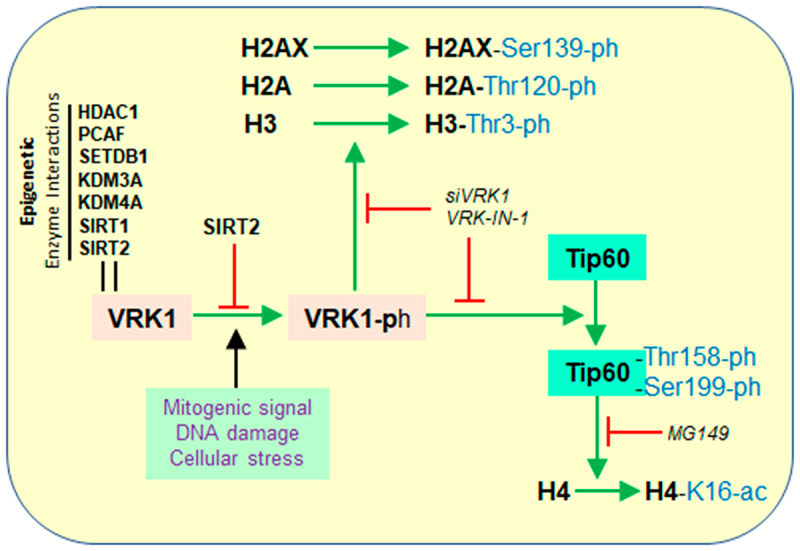
Effect of VRK1 on different histones in response to different types of stimulation and its interactions with different types of enzymes performing histone posttranslational modifications. VRK-IN-1: VRK inhibitor 1. MG149: Tip60 inhibitor. HDAC1: histone deacetylase 1. PCAF: (P300/CBP-Associated factor or KAT2B), SETDB1: SET domain bifurcated histone lysine methyltransferase 1 or KMT1E. KDM3A: Lysine demethylase 3A or JHMD2A. KDM4A: Lysine demethylase 4A. SIRT1: NAD-Dependent protein deacetylase sirtuin-1. SIRT 2: NAD-Dependent protein deacetylase sirtuin-2.

The different patterns of histone epigenetic modifications are associated with several types of biological functions [10,11], including transcription, replication, recombination, and DNA damage responses to pathological situations such as cancer [12–14] and neurological diseases [31]. Epigenetic modifications alter nucleosomes, which can have different conformations and functional effects on chromosomal dynamics [32,33]. In addition to kinases, chromatin organization is also regulated by other enzymes that perform additional types of histone epigenetic modifications, such as acetylation, methylation, and ubiquitination [17]. These epigenetic posttranslational modifications of histones are indirectly regulated by VRK1, and these epigenetic modifications are altered by either VRK1 depletion or by VRK-IN-1 [34], a specific inhibitor of this kinase [35]. VRK1 can only play an additional role in the regulation of other histone epigenetic modifications through indirect mechanisms, in which these epigenetic enzymes are likely targets to be regulated and coordinated by VRK1 [27,34]. VRK1, in addition to histone phosphorylation, indirectly regulates histone epigenetic patterns through either phosphorylation or direct interaction with different types of epigenetic enzymes (Figure 1). VRK1 stably interacts with several epigenetic enzymes, such as HDAC1, PCAF, SETDB1, KDM3A, and KDM4A [27], which belong to different families, but their effects are mostly unknown (Figure 1). VRK1 directly phosphorylates Tip60/KAT5 at Thr158 and Ser199 in response to DNA damage [25,26] promoting Tip60 translocation from the nucleoplasm to chromatin, and the specific acetylation of H4K16 [25,26]. The interaction between VRK1 and SIRT2 inhibits the kinase activity of VRK1 [27], and facilitates the deacetylation of H4K16ac by SIRT2 [36,37]. Deacetylation of H4K16ac by SIRT1 impairs the recruitment of SMARCAD1 to DNA double-strand breaks [38]. The regulation of histone PTMs by VRK1 indicates that this kinase is a master regulator of chromatin organization and is associated with several nuclear functions [27,34].

Epigenetic modifications alter nucleosomes, which can have different conformations and functional effects on chromosomal dynamics [32,33]. In addition to phosphorylation, chromatin organization is also regulated by other enzymes that perform additional types of histone epigenetic modifications, such as acetylation, methylation, and ubiquitination [17]. These epigenetic posttranslational modifications of histones can be indirectly regulated by VRK1, and consequently histone epigenetic modifications can be altered by either VRK1 depletion or by VRK-IN-1 [34], a specific inhibitor of this kinase [35]. Loss of VRK1 by depletion, or its inhibition, functionally mimics the effect of different types of epigenetic inhibitors targeting histone acetyl (KAT) and methyl transferases (KMT), as well as histone deacetylases (HDAC) and demethylases (KDM) [34]. Depletion of VRK1, or its inhibition with VRK-IN-1, causes a switch in the patterns of H3K4, H3K9, and H3K27 post-translational modifications [34]. VRK1 depletion/inhibition causes the loss of H3K9/H3K27 acetylation, and permits their methylation. This effect is similar to that of the p300 inhibitor C646 and KDM inhibitors, such as iadademstat (ORY-1001) or JMJD2 inhibitor [34]. Alternatively, HDAC inhibitors (selisistat, panobinostat, and vorinostat) and KMT inhibitors (tazemetostast and chaetocin) have the opposite effect of VRK1 depletion or inhibition, and cause an increase in H3K9ac and a decrease in H3K9me3 [34]. VRK1 depletion also impairs the H4K20me2 modification, which is required for the recruitment of 53BP1 in DNA repair by the non-homologous end-joining (NHEJ) pathway [39], functioning in a manner similar to KDM inhibitors such as chaetocin or tazemetostat [40]. Alternatively, the kinase activity of VRK1 can be inhibited by its interaction with another epigenetic enzyme, as is the case of a direct protein interaction between VRK1 and SIRT2, which inhibits the kinase and alters the pattern epigenetic posttranslational modifications of histones [27].

## VRK1 gene transcription and cell cycle progression

**Figure 2.**
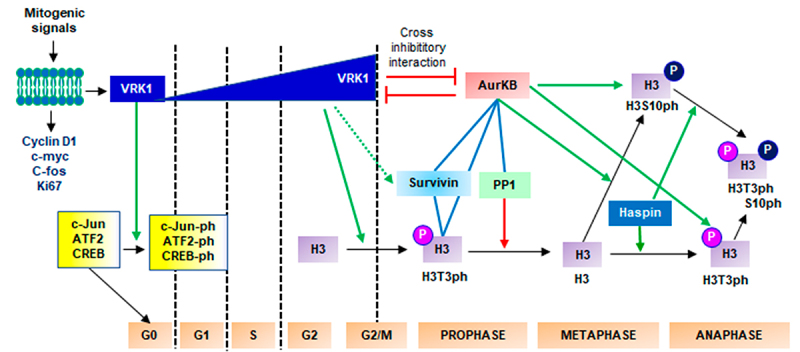
VRK1 in proliferation and cell cycle progression. Basal VRK1 expression is increased in response to mitogenic signals. Arrows: indicate direct phosphorylations (green) or dephosphorylation (red). Direct protein interactions (blue lines).

Mitogenic signals, such as growth factors induce *VRK1* gene expression, which expression parallels that of *MYC*, *FOS, CCND1* (cyclin D1) and Ki67 in proliferative/mitogenic responses [41,42]. However, VRK1 is not active unless it is phosphorylated by autophosphorylation [43], or indirectly by the BRAF, MEK, and ERK, a pathway that is also activated in response to mitogenic signaling. Phosphorylation of VRK1 kinase is blocked by MAPK signaling inhibitors. The activated VRK1 directly phosphorylates several transcription factors implicated in cell cycle progression, which includes c-Jun [44], ATF2 [45] and CREB [46] and p53 [43,44,47] (Figure 2). VRK1 depletion blocks cell cycle progression [42]. In stratified epithelia, the highest VRK1 expression was detected in a few individual cells in the basal layer, the one that has started to divide; in the next layer, all cells are positive and have high levels. As epithelial cell terminal differentiation progresses in the epithelium, the level of VRK1 is progressively reduced [41,48].

VRK1 plays different roles in the cell cycle (Figure 2), from entry into the cell cycle to the end of mitosis. Initially, the expression of *VRK1* is upregulated in response to mitogenic signals, such as the addition of growth factors to serum-deprived cells for entry into cell proliferation and the G0/G1 transition [42,49]. In response to mitogenic signals, VRK1 overexpression correlates with c-Myc, FOS, and Ki67 expression and their increased levels [24,41,42,50–52], phosphorylation of retinoblastoma (RB), and the expression of cyclin D1 [22,42,46]. The expression of VRK1 is enhanced by mitogenic signals, and VRK1 is required for G0/G1 and [42,49] and G2/M progression [23,41]. Depletion of VRK1 [42] or targeting with specific aptamers [53] blocks *CCND1* gene expression, G0 exit, and cell proliferation. Upstream activation of *VRK1* gene expression by mitogenic signals is blocked by inhibitors targeting sequential steps in the MAPK signaling pathway, such as BRAF, MEK, and ERK. VRK1 activation is associated with G0 exit [42], as well as with the levels of cytosolic Vaccinia-related Kinase 2 (VRK2), which regulates the Erb2 pathway [54,55]. In chromatin, activated VRK1 phosphorylates H3 at Thr3, which is required for G0 exit [41,42] and transcription activation [41,44,45,56] in response to mitogenic signals. This phosphorylation of H3 at Thr3 is also necessary for the G1/S transition [41] and G2/M progression [23,24,57]. The translational efficiency of VRK1 mRNA is increased by its binding to the 3’-untranslated region of hnRNPA1 [58], and thus potentiates the expression of *CCND1* (cyclin D1) mediated by phosphorylation of CREB [46], which promotes lung cancer tumor cell proliferation [58]. Furthermore, hnRNPA1 directly binds to 3’-untranslated VRK1 mRNA, which contributes to its translation and tumor cell proliferation [58]. VRK1 phosphorylates hnRNPA1 and facilitates its binding of hnRNPA1 to telomeric single-stranded DNA and telomerase RNA, which stimulates telomerase activity [59].

The hnRNP A1 protein is phosphorylated by VRK1, promoting its binding to telomerase RNA and telomeric ssDNA and enhancing the telomerase reaction [59]. Thus, VRK1 deficiency induces shortening of telomeres and aberrant organization in mouse male germ cells [60], which is consistent with the male sterility detected in VRK1 deficient mice [61].

During the G2/M transition [23] and meiotic progression [62], H3Thr3ph is read by survivin and interacts with Aurora B (AURKB) [63]. In mitosis, this transient interaction between VRK1 and AURKB cross inhibits their kinase activities [24]. Active VRK1 is dephosphorylated by the mitogen-activated protein kinase phosphatase 2 (MKP2) phosphatase, preventing the rephosphorylation of H3 [24,57,64], which is later rephosphorylated by haspin during mitotic progression [65], which facilitates the recruitment of the chromosomal passenger complex (CPC) to centromeres, of which AURKB is a catalytic component [66] that phosphorylates H3Ser10 [67]. In *Drosophila melanogaster*, NHK-1 is required for mitotic [1] and meiotic [68,69] progression. In a murine gene-trap model of VRK1, deficiency in levels of VRK1 causes male sterility by impairing the formation of spermatogonia [61], and female sterility by causing defective folliculogenesis [70] and interfering with the progression of oogenesis [62,71].

For a correct cell division, the fragmentation of some intracellular membranes is necessary for its redistribution into daughter cells, and in this context VRK1 also plays a role. In G2/M, Golgi fragmentation, which is regulated by serine-threonine kinases, is required for its redistribution into daughter cells during mitosis. Golgi fragmentation is activated by MEK1 [72], ERK1 [73] and Plk3 [74]. Golgi fragmentation is induced by mitogenic signals that are blocked downstream of Plk3 by a kinase-dead VRK1(K179E) as well as by the VRK1 knockdown, kinase-dead Plk3, or PD98059 MEK1 inhibitor [75]. Plk3 directly interacts with VRK1 to form a stable complex, and both kinases colocalize with giantin in granules, resulting from Golgi fragmentation in the cytoplasm [75]. Plk3 is upstream and phosphorylates the C-terminal region of VRK1 at Ser342, but VRK1 does not phosphorylate Plk3 [75]. This specific phosphorylation of VRK1 is necessary for Golgi fragmentation, mimicked by the mutation to aspartic acid (S342D), but its mutation to alanine (S342A) blocks Golgi fragmentation. These data indicate that VRK1 phosphorylation at Ser342 is required for cell cycle progression. MEK1-Plk3-VRK1 constitutes a sequential module in the regulation of Golgi fragmentation during cell cycle and mitotic progression [75].

In cells with low VRK1 levels, some VRK1 functions are compensated by the expression of a splice variant of VRK2, which is cytosolic and anchored to the endoplasmic reticulum membranes [76]. This VRK2 variant is generated by alternative splicing and lacks the VRK2 C-terminal region, which contains an endoplasmic reticulum signal. This shorter VRK2 variant is located in the nucleus [76], and partially shares several interaction and phosphorylation partners with VRK1 [77].

Moreover, deficiency in VRK1 levels causes telomere shortening and abnormal arrangement, which triggers the activation of DNA damage responses in male murine germ cells [59]. In mice, deficiency of VRK1 causes male and female infertility due to a progressive loss of spermatogonia [61] and impaired oogenesis by defects in folliculogenesis [70] and meiotic recombination [62].

## VRK1 in DNA damage responses

**Figure 3.**
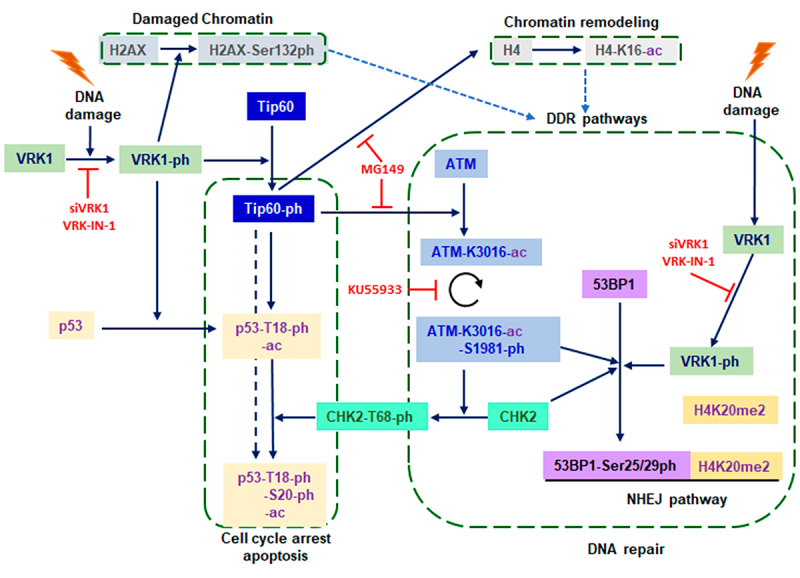
VRK1 and DNA damage responses. Diagram illustrating the main pathways triggered by DNA damage in the response to different types of DNA damage, and the implication of VRK1 in different steps of the process, from altered chromatin to repair pathways.

Local DNA damage alters chromatin organization [78,79]. The implication of VRK1 in DNA damage response (DDR) is associated with several functions, chromatin and epigenetic modifications [27,34], and DDR pathways. The organization of chromatin conditions accessibility and sensitivity to different types of DNA damage. The induction of DNA damage by doxorubicin or oxidative stress alters the nuclear phosphoproteome, which is enriched in histone modification proteins and chromatin-associated proteins that are impaired by VRK1 depletion [80,81]. These observations are consistent with the role of VRK1 in biological processes that require chromatin remodeling (Figure 3).

VRK1 kinase activity is triggered by DNA damage independent of its type, including oxidative stress, alkylating drugs, ionizing radiation, doxorubicin, olaparib, temozolomide and replication block [28,82–85]. Moreover, VRK1 functional downregulation by mutation or inhibition sensitizes tumor cells to genotoxic treatments [4,31,39,82,86]. All of these types of DNA damage, which locally alter chromatin, induce the activating autophosphorylation of VRK1, which phosphorylates different types of targets, including epigenetic enzymes, DDR proteins, and transcription factors. These proteins are regulated by VRK1 and their functions impaired by VRK1 depletion or inhibition [5,28,82,85].

In the DNA damage response (DDR), VRK1 directly phosphorylates histone H2AX (γH2AX) [28], NBS1 in Ser343 [85], 53BP1 in Ser25/29, and is independent of ATM [82]. VRK1 depletion impairs the activation of ATM, CHEK2, and DNA-PK in response to radiation [82], doxorubicin [84,87] and olaparib [88] treatments. The role of VRK1 in DDR is upstream of ATM and DNA-PK [82]. These proteins form part of the sequential steps in the DNA damage response, which is functional in both proliferating and cell-cycle arrested cells [28,82,84,85].

Alterations in DDR pathways have been implicated in cancer and neurological diseases. High levels of VRK1 confer resistance to different treatments based on DNA damage; thus, VRK1 overexpression has been associated with poorer prognosis in some types of cancer, including multiple myeloma and lung cancer [4]. Furthermore, several neurological, neurodegenerative, and motor neuron diseases (MND) are associated with alterations in DNA damage repair (DDR) pathways [89–91], as well as alterations in Cajal bodies and its associated proteins [92–94]. Among the proteins involved in the pathogenesis of neurological diseases are ATM, NBS1, and CHEK2, all of which are involved in DDR [95–97].

## VRK1 and p53 mediated responses

The p53 protein regulates the cell cycle, DNA damage responses, and apoptosis [98,99]. The immediate cell response to DNA damage requires the phosphorylation of p53. VRK1 participates in the early responses to DNA damage. This mechanism detects and reacts to local alterations in the chromatin structure induced by DNA damage. The VRK1 protein directly interacts to form a complex with p53 in the basal state [56,100]. This interaction occurs through the p53 N-terminal regulatory domain, and frequent DNA-contact mutants of p53, such as R273H, R248H or R280K, do not disrupt the VRK1-p53 complex [43,101]. Therefore, the basal VRK1-p53 complex functions as an early warning system for immediate cellular responses to cellular stress and DNA damage. In response to different types of DNA damage, VRK1 directly phosphorylates p53 in Thr18 [43,56,102]. This specific phosphorylation triggers a p53 functional switch from binding to ubiquitin ligases such as mdm2 to transcription factors [103,104]. This specific phosphorylation at Thr18 distorts the p53 hydrophobic alpha helix, preventing the p53 interaction with the mdm2 hydrophobic pocket; thus, p53 degradation by ubiquitination is impaired, which facilitates p53 protein stabilization and accumulation [56], and permits its interaction with DNA and gene transcription [104]. This specific p53 phosphorylation functions as a switch between protein degradation and gene transcription [98,104,105]. The activation and stabilization of p53 permits the induction of cell cycle arrest and facilitates DNA repair or cell death when DNA damage is excessive. In case, the stress or damage is solved in the initial response, the p53 accumulation is reversed by a novel p53-dependent activation of autophagy that removes its activating VRK1 [106], thus facilitating p53 dephosphorylation and its downregulation by mdm2 to bring p53 back to basal levels [100,102,106,107]. In many types of cancers with p53 mutations, this autoregulatory loop is defective, and facilitates cancer cell progression and resistance to treatments [48,102].

## VRK1, BAF (barrier-to-autointegration) and nuclear envelope

**Figure 4.**
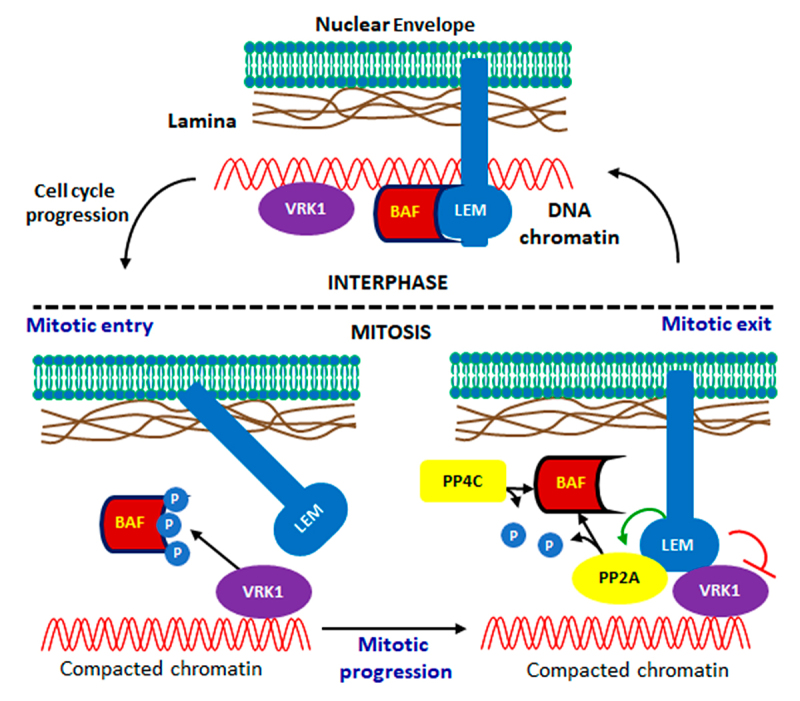
Regulation of BAF by VRK1 in mitotic progression. Red and green arrows indicate repression or activation respectively. Black arrows indicate the activity of VRK1 kinase and the PP2A and PP4C phosphatases.

The BAF (BANF1, barrier-to-autointegration factor 1) protein interacts with LEM domain proteins (LAP2, emerin, and MAN1) in *C. elegans* [108] and humans [109] (Figure 4). The BAF protein bridges double-stranded DNA in a highly ordered nucleoprotein complex, but its interaction with the DNA sequence is nonspecific [110]. BAF is required to segregate and enclose chromosomes within the nuclear envelope, and also assembles the nuclear lamina [111]. BAF participates in the formation of the nuclear envelope (NE), and NE defects occur prior to chromatin alterations [108] and interact with core histones [112]. The phosphorylation of BAF by VRK1 [113] regulates its interaction with the nuclear envelope [114], and facilitates chromosome attachment and chromatin organization, two processes that are altered by VRK1 depletion [108]. In addition, a fraction of BAF is associated with centromeres and the control of mitotic progression [115], which is also impaired by VRK1 depletion [24] or VRK1 pathogenic variants [31].

In mammals, non-phosphorylated BAF mediates the interaction of chromatin with the inner nuclear membrane in interphase and facilitates the attachment of chromosomes to the nuclear envelope [116], the binding of emerin to the inner nuclear envelope [117], and assembly of the nuclear lamina [111] (Figure 4). BAF compacts chromatin via a looping mechanism [118]. VRK1 is necessary for the expression of BAF in cell cycle progression [119]. The VRK1 protein directly interacts with and phosphorylates BAF in Ser4 and Thr3 [71,113,114,120,121], preventing the interaction of emerin with the nuclear envelope and DNA, and facilitating BAF detachment from the nuclear envelope [113]. Therefore, the dephosphorylation of BAF is necessary for the reassembly of the nuclear envelope at the end of mitosis, which requires LEM4/ANKLE2, an inner nuclear membrane protein in mammals, and implicates a coordination of PP2A phosphatase and VRK1, a function that is conserved from worms to humans [122]. LEM4/ANKLE2 inhibits VRK1 and controls the dephosphorylation of BAF by protein phosphatase 2A (PP2A), thereby facilitating nuclear envelope assembly [122]. In chromatin, mitogen-activated protein kinase phosphatase 2 (MKP2) interacts with VRK1 and suppresses histone H3 [64]. In the nuclear envelope, LEM4 promotes the dephosphorylation of BAF during mitotic exit [123]. Furthermore, SIRT2 interacts with LEM4 to inhibit its acetylation [124] and prevents BAF phosphorylation, facilitating its detachment from the nuclear envelope. In a zebrafish experimental model, ANKLE2 deficiency caused microcephaly, spermatogenic defects [125] and motor dysfunction [126], symptoms that have also been reported in patients with rare VRK1 mutations [127].

The extensive flexibility of the BAF N-terminal helix α1 and loop α1α2 is strongly reduced in diphosphorylated BAF due to interactions between the phosphorylated residues and the positively charged C-terminal helix α6. These regions are involved in DNA and lamin A/C-binding. BAF phosphorylation causes a major loss of its affinity for dsDNA, but does not impair its binding to the lamin A/C Ig fold domain and emerin nucleoplasmic region, which leaves open the question of the coordination of these protein interactions and their functions [120].

BAF bridges DNA using two pairs of helix-hairpin-helix motifs, which are located on opposite surfaces of the BAF dimers [128]. BAF interaction with DNA is also involved in the regulation of DNA double-strand break (DSB) repair by the inhibition of DNA-PK, which is required for the choice of DSB repair pathway [129]. BAF relocalizes from the nuclear envelope to sites of double-strand breaks [129]. This effect is similar to that of VRK1 depletion, which impairs DNA-PK activity in response to DNA damage [82]. Oxidative stress induces the binding of BAF to Poly-(ADP-Ribose) polymerase 1 (PARP1) causing the inhibition of PARP1 auto-ADP-ribosylation and thus a defective repair of oxidative lesions, particularly in cells with high levels of BAF [129,130]. VRK1 downregulation reduces the expression of BAF and inhibited the proliferation and migration of esophageal squamous cell carcinoma (ESCC) [121]. Depletion of human VRK1 results in aberrant nuclear architecture, in which nuclear BAF is elevated and sustained interaction with its partners is likely to account for aberrant NE organization [114]. This disruption of the nuclear envelope (NE) by inactivation of BAF causes an accumulation of tau protein and is a likely initiating event in the pathogenesis of tauopathies [131].

## VRK1, coilin and Cajal bodies

**Figure 5.**
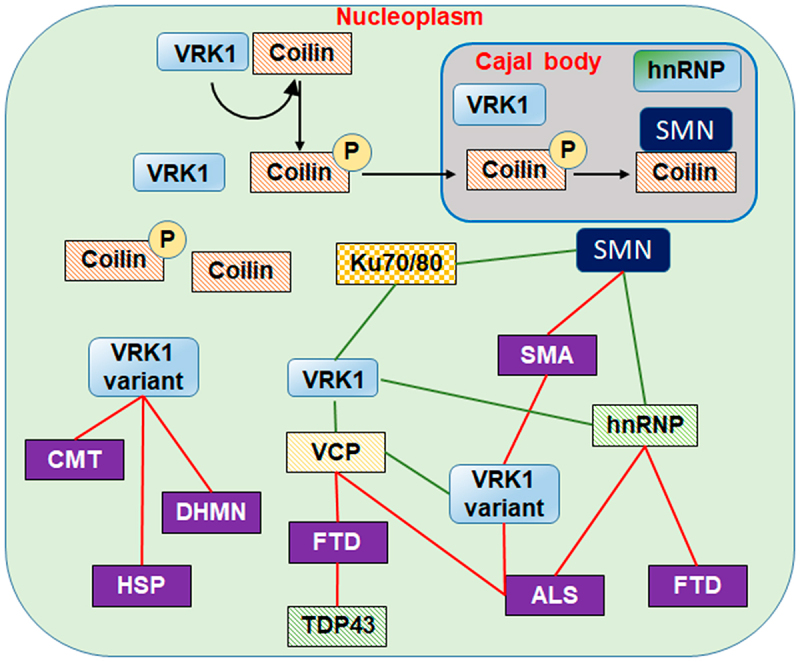
VRK1, Cajal bodies and neurological diseases altered by VRK1 variants. VRK1 phosphorylates coilin that is translocated to the Cajal bodies, where it interacts with SMN. Alterations in the process by pathogenic variants of VRK1 impair different nuclear proteins and lead to very severe neurological diseases. SMA: spinal muscular atrophy. CMT: Charcot-Marie-tooth. HSP: hereditary spastic paraplegia. DHMN: distal hereditary motor neuropathies. FTD: front- temporal degeneration. ALS: amyotrophic lateral sclerosis. VCP (valosin containing protein). Ku70/80 (ATP dependent helicases). VRK1 and VCP mutant proteins are associated with amyotrophic lateral sclerosis (ALS). Red lines: disease. Green lines: interactions.

Cajal bodies are nuclear structures identified by Ramón y Cajal in 1903 [132]. These structures are assembled on coilin as its scaffold protein [133] on which different RNP complexes are assembled [134,135] with different types of RNA, including spliceosomal nuclear RNA (snRNA) and small nucleolar RNAs (snoRNAs), by forming small nuclear ribonucleoprotein particles (snRNPs) [135] (Figure 5). These RNP particles are involved in different functions such as splicing, ribosome biogenesis, and telomere maintenance [136].

Coilin regulates the formation and organization of Cajal bodies and functions as a scaffold protein for the assembly of RNP complexes [92,135]. VRK1 directly phosphorylates coilin at Ser184 and facilitates its assembly and stability in CBs organization [9] (Figure 5). This specific phosphorylation of coilin at Ser-184 prevents Cajal body disassembly [8,137]. Non-phosphorylated coilin leads to CBs disintegration, its ubiquitination within nuclei, and its transport to the cytosol, where coilin is degraded in the proteasome [9]. This effect of non-phosphorylated coilin is prevented by either blocking its nuclear export with leptomycin or by inhibiting its proteasomal degradation after its export to the cytosol [9]. Another potential role of coilin is due to its involvement on the organization of the Histone locus bodies (HLBs), which are biomolecular condensates that assemble replication-dependent histone genes in animal cells [138]. HLBs share components with Cajal bodies [136,139,140], and contain factors that are required for the processing of histone pre-mRNAs [141]. However, the role of coilin and its regulation by VRK1 in HLB remains to be characterized.

**Figure 6.**
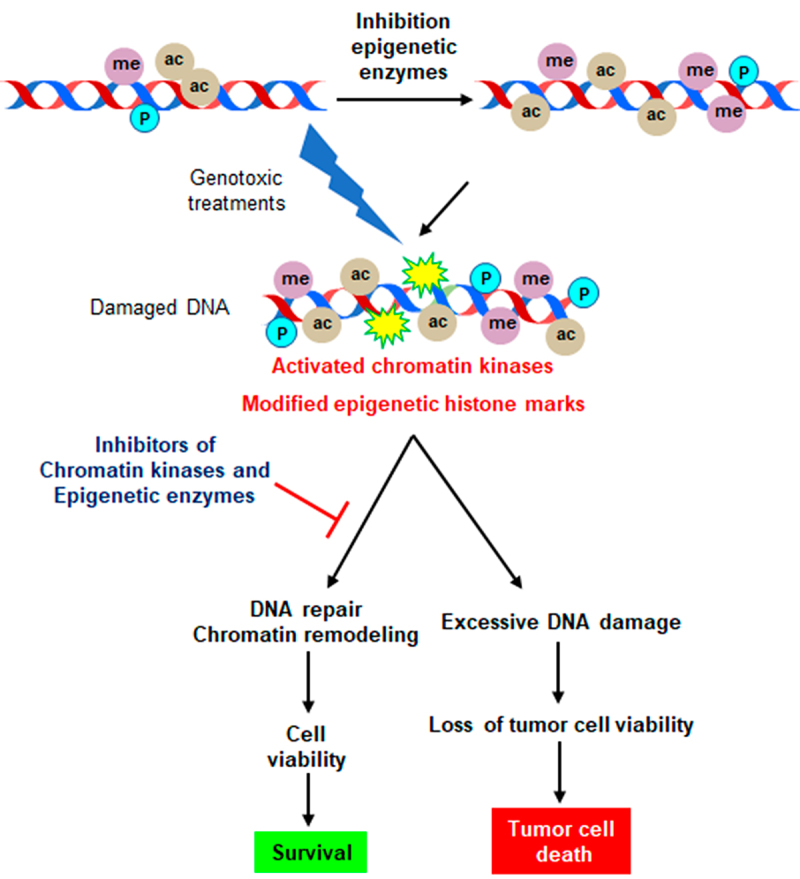
Targeting of epigenetic modifiers in cancer treatment. The combined drug targeting facilitates a switch from repair mechanisms toward damage accumulation. Different drugs combination in synthetic lethality strategies might facilitate a reduction of individual drug dose, and thus their toxicity, while promoting damage accumulation leading to tumor cell death.

Rare homozygous or compound heterozygous human VRK1 variants were associated with distal hereditary neuropathies and motor neuron diseases [31] (Figure 6). These VRK1 variants alter Cajal body assembly, and the assembly and stability of RNPs complexes, which are the main factors underlying Cajalopathies [31]. Coilin interacts with multiple proteins [142]. Thus, VRK1 variants might interact differently with protein complexes assembled on coilin and consequently display some differences in their pathological effects. These pathogenic VRK1 variants associated with neurological diseases cause an altered assembly of Cajal bodies [31,86,143,144], and some also have an altered action potential [145]. VRK1 pathogenic variants mainly affect neurons with long axons, such as motor neurons. Among the diseases associated with these VRK1 variants are spinal muscular atrophy (SMA), amyotrophic lateral sclerosis (ALS), distal hereditary motor neuropathies (dHMN), and Charcot-Marie-Tooth (CMT) [31]. In addition, mutations in hnRNPA1 have also been reported in patients with amyotrophic lateral sclerosis [146] and myopathies [147].

## VRK1 in cancer, targeting VRK1 for novel cancer therapies

High levels of VRK1 facilitate tumor cell proliferation [42,58], resistance to treatments based on DNA damage [4,5,85], and epithelial-mesenchymal transition in breast cancer cells [148,149]. High levels of wild-type VRK1 expression are associated with poorer prognosis in many types of cancer [4] including multiple myeloma [150], lung cancer [4,41,58,151], head and neck squamous cell carcinoma [48], esophageal cancers [152], gastric cancer [153], liver [154], colon [155], breast [84,149,156], hepatocellular carcinomas [154], Wilms tumors [157] and gliomas [158–160].

The identification of druggable targets in chromatin regulation could potentially be exploited in synthetic lethality strategies. In this context, kinases and other epigenetic modifiers are likely targets. The structure of the VRK1 catalytic site is atypical and is thus insensitive to current types of kinase inhibitors [161–164]. The mechanism of the VRK1-ATP complex is not well understood [165]. Thus, most information regarding its loss of function has been obtained by experiments based on the depletion of VRK1, which has been shown to impair cell proliferation [42] and response to DNA damage [4,5], and lately by the use of specific VRK1 inhibitors [27,34,35,166]. Depletion of VRK1 sensitizes cells to genotoxic cancer treatments. Initially, VRK1 depletion sensitized cells to DNA damage induced by ionizing radiation or doxorubicin. DNA damage detected by the induction of ionizing radiation (IR) or doxorubicin caused double-strand breaks detected by the formation of yH2AX [28,84] and 53BP1. VRK1 depletion in combination with either ionizing radiation (IR) [88] or doxorubicin [84] resulted in a reduction in the dose required to achieve a similar effect.

VRK1 depletion or inhibition has an important effect on epigenetic posttranslational modifications of histones, and as an indirect epigenetic modifier, is also a potential target for the development of novel therapeutic combinations in cancer [13,167,168]. VRK1 depletion promotes the loss of H3K9ac and H3K27ac, which are associated with gene transcription and cell proliferation, and facilitates trimethylation of H3K9 and K27 [34]. VRK1 depletion also impairs the accumulation of H4K20m2 [39], similar to KMT inhibitors [40], which is necessary for the recruitment of 53BP1 in the non-homologous end joining (NHEJ) DNA damage repair pathway [169,170], thus sensitizing tumor cells to genotoxic treatments. VRK1 depletion also impairs the DNA damage response triggered by treatment with olaparib [39], a PARP inhibitor, and ionizing radiation [39,88]. Depletion of VRK1 sensitizes glioblastoma (GBM) cells to PARP inhibitors, such as olaparib, by facilitating DNA damage in tumor cells and reducing their viability [39]. In GBM, VRK1 depletion impaired the formation of H4K20me2 that is required for the recruitment of 53BP1 to damaged DNA sites in glioblastoma cells treated with temozolomide and olaparib [39].

The structure of VRK1 indicates that its kinase domain has characteristics that make it a candidate for the development of specific inhibitors [162,163], consistent with the lack of effect of inhibitors targeting different kinase families [164]. Recently, a specific VRK1/VRK2 inhibitor (VRK-IN-1) was developed [35]. This inhibitor impairs the phosphorylation of VRK1 targets, such as histone H3 and Tip60/KAT5 [27,34], altering epigenetic posttranslational modifications of chromatin in both basal and DNA damage responses [27,34]. VRK1 depletion or its inhibition with VRK-IN-1 also facilitate interferon-mediated immune responses [171,172]. Thus, it is a starting point for the development of better inhibitors targeting VRK1, which can be used in combination with additional inhibitors directed to proteins in other signaling pathways, and which in cooperation can facilitate tumor cell death at lower drug doses in synthetic lethality strategies [39]. Targeting VRK1 in glioblastoma cells, neuroblastomas, and pediatric gliomas, in which VRK2 has been eliminated or silenced by VRK2-promoter methylation cells [159,173], is a potential synthetic lethality strategy in these tumors. The combination of drugs targeting different families of epigenetic post-translational modification enzymes can be a useful strategy for cancer treatment (Figure 6).

## Perspective

The roles played by VRK1 in the context of several different nuclear functions can have important implications for the understanding of major diseases such as cancer and neurological diseases. High levels of VRK1 promote tumor cell expansion by enhancing cell proliferation and resistance to treatment. In the context of cancer, the inhibition of VRK1 activity has the potential to be a vulnerability in tumor cells and thus become a target for use in novel synthetic lethality strategies in cancer treatment. Regarding neurological diseases, the implication of rare pathogenic VRK1 variants associated with very severe motor neuron diseases can be useful for a better characterization of these diseases and contribute to improved diagnosis and the design of specific new approaches for their clinical management.

## Data Availability

Data sharing is not applicable to this article as no new data were created or analyzed in this study.

## Abbreviations

VRK1: vaccinia related kinase 1
NHK-1: nucleosomal histone kinase-1
CB: Cajal body
ALS: amyotrophic lateral sclerosis
SMA: Spinal muscular atrophy
CMT: Charcot-Marie-Tooth
dHMN: distal hereditary motor neuropathy.
RNP: Ribonucleoprotein.
DDR: DNA damage response

## References

[1] Cullen CF, Brittle AL, Ito T, et al. The conserved kinase NHK-1 is essential for mitotic progression and unifying acentrosomal meiotic spindles in drosophila melanogaster. J Cell Bio. 2005;171(4):593–18. doi: 10.1083/jcb.200508127 16301329 PMC2171570

[2] Manning G, Whyte DB, Martinez R, et al. The protein kinase complement of the human genome. Science. 2002;298(5600):1912–1934. doi: 10.1126/science.1075762 12471243

[3] Nichols RJ, Traktman P. Characterization of three paralogous members of the mammalian vaccinia related kinase family. J Biol Chem. 2004;279(9):7934–7946. doi: 10.1074/jbc.M310813200 14645249

[4] Campillo-Marcos I, García-González R, Navarro-Carrasco E, et al. The human VRK1 chromatin kinase in cancer biology. Cancer Lett. 2021;503:117–128. doi: 10.1016/j.canlet.2020.12.032 33516791

[5] Campillo-Marcos I, Lazo PA. Implication of the VRK1 chromatin kinase in the signaling responses to DNA damage: a therapeutic target? Cell Mol Life Sci. 2018;75(13):2375–2388. doi: 10.1007/s00018-018-2811-2 29679095 PMC5986855

[6] Alfert A, Moreno N, Kerl K. The BAF complex in development and disease. Epigenet Chromatin. 2019;12(1):19. doi: 10.1186/s13072-019-0264-y PMC6427853 30898143

[7] Kakarougkas A, Ismail A, Chambers AL, et al. Requirement for PBAF in transcriptional repression and repair at DNA breaks in actively transcribed regions of chromatin. Mol Cell. 2014;55(5):723–732. doi: 10.1016/j.molcel.2014.06.028 25066234 PMC4157577

[8] Sanz-Garcia M, Vazquez-Cedeira M, Kellerman E, et al. Substrate profiling of human vaccinia-related kinases identifies coilin, a cajal body nuclear protein, as a phosphorylation target with neurological implications. J Proteomics. 2011;75(2):548–560. doi: 10.1016/j.jprot.2011.08.019 21920476

[9] Cantarero L, Sanz-Garcia M, Vinograd-Byk H, et al. VRK1 regulates cajal body dynamics and protects coilin from proteasomal degradation in cell cycle. Sci Rep. 2015;5:10543. doi: 10.1038/srep10543 26068304 PMC4464288

[10] Kouzarides T. Chromatin modifications and their function. Cell. 2007;128(4):693–705. doi: 10.1016/j.cell.2007.02.005 17320507

[11] Bannister AJ, Kouzarides T. Regulation of chromatin by histone modifications. Cell Res. 2011;21(3):381–395. doi: 10.1038/cr.2011.22 21321607 PMC3193420

[12] Zhao Z, Shilatifard A. Epigenetic modifications of histones in cancer. Genome Biol. 2019;20(1):245. doi: 10.1186/s13059-019-1870-5 31747960 PMC6868810

[13] Nebbioso A, Tambaro FP, Dell’aversana C, et al. Cancer epigenetics: moving forward. PLOS Genet. 2018;14(6):e1007362. doi: 10.1371/journal.pgen.1007362 29879107 PMC5991666

[14] Audia JE, Campbell RM. Histone modifications and cancer. Cold Spring Harb Perspect Biol. 2016;8(4):a019521. doi: 10.1101/cshperspect.a019521 27037415 PMC4817802

[15] MacDonald JL, Tharin S, Hall SE. Epigenetic regulation of nervous system development and function. Neurochem Int. 2022;152:105249. doi: 10.1016/j.neuint.2021.105249 34826529

[16] Kumar R, Deivendran S, Santhoshkumar TR, et al. Signaling coupled epigenomic regulation of gene expression. Oncogene. 2017;36(43):5917–5926. doi: 10.1038/onc.2017.201 28650470

[17] Tessarz P, Kouzarides T. Histone core modifications regulating nucleosome structure and dynamics. Nat Rev Mol Cell Biol. 2014;15(11):703–708. doi: 10.1038/nrm3890 25315270

[18] Valbuena A, Sanz-Garcia M, Lopez-Sanchez I, et al. Roles of VRK1 as a new player in the control of biological processes required for cell division. Cell Signal. 2011;23(8):1267–1272. doi: 10.1016/j.cellsig.2011.04.002 21514377

[19] Nikalayevich E, Ohkura H. The NuRD nucleosome remodelling complex and NHK-1 kinase are required for chromosome condensation in oocytes. J Cell Sci. 2015;128(3):566–575. doi: 10.1242/jcs.158477 25501812 PMC4311133

[20] Shin J, Chakraborty G, Bharatham N, et al. NMR solution structure of human vaccinia-related kinase 1 (VRK1) reveals the C-terminal tail essential for its structural stability and autocatalytic activity. J Biol Chem. 2011;286(25):22131–22138. doi: 10.1074/jbc.M110.200162 21543316 PMC3121357

[21] Budziszewski GR, Zhao Y, Spangler CJ, et al. Multivalent DNA and nucleosome acidic patch interactions specify VRK1 mitotic localization and activity. Nucleic Acids Res. 2022;50(8):4355–4371. doi: 10.1093/nar/gkac198 35390161 PMC9071384

[22] Aihara H, Nakagawa T, Mizusaki H, et al. Histone H2A T120 phosphorylation promotes oncogenic transformation via upregulation of cyclin D1. Mol Cell. 2016;64(1):176–188. doi: 10.1016/j.molcel.2016.09.012 27716482

[23] Kang TH, Park DY, Choi YH, et al. Mitotic histone H3 phosphorylation by vaccinia-related kinase 1 in mammalian cells. Mol Cell Biol. 2007;27(24):8533–8546. doi: 10.1128/MCB.00018-07 17938195 PMC2169395

[24] Moura DS, Campillo-Marcos I, Vazquez-Cedeira M, et al. VRK1 and AURKB form a complex that cross inhibit their kinase activity and the phosphorylation of histone H3 in the progression of mitosis. Cell Mol Life Sci. 2018;76(14):2591–2611. doi: 10.1007/s00018-018-2746-7 PMC6003988 29340707

[25] Garcia-Gonzalez R, Morejon-Garcia P, Campillo-Marcos I, et al. VRK1 phosphorylates Tip60/KAT5 and is required for H4K16 acetylation in response to DNA damage. Cancers (Basel). 2020;12(10):2986. doi: 10.3390/cancers12102986 33076429 PMC7650776

[26] García-González R, Monte-Serrano E, Morejón-García P, et al. The VRK1 chromatin kinase regulates the acetyltransferase activity of Tip60/KAT5 by sequential phosphorylations in response to DNA damage. Biochim Biophys Acta, Gene Regul Mech. 2022;1865(8):194887. doi: 10.1016/j.bbagrm.2022.194887 36280132

[27] Monte-Serrano E, Lazo PA. VRK1 kinase activity modulating histone H4K16 acetylation inhibited by SIRT2 and VRK-IN-1. Int J Mol Sci. 2023;24(5):4912. doi: 10.3390/ijms24054912 36902348 PMC10003087

[28] Salzano M, Sanz-Garcia M, Monsalve DM, et al. VRK1 chromatin kinase phosphorylates H2AX and is required for foci formation induced by DNA damage. Epigenetics. 2015;10(5):373–383. doi: 10.1080/15592294.2015.1028708 25923214 PMC4623420

[29] Fernandez-Capetillo O, Lee A, Nussenzweig M, et al. H2AX: the histone guardian of the genome. DNA Repair. 2004;3(8–9):959–967. doi: 10.1016/j.dnarep.2004.03.024 15279782

[30] Maeda K, Yoneda M, Nakagawa T, et al. Defects in centromeric/pericentromeric histone H2A T120 phosphorylation by hBUB1 cause chromosome missegregation producing multinucleated cells. Genes Cells. 2018;23(10):828–838. 30112853 10.1111/gtc.12630

[31] Lazo PA, Morejón-García P. VRK1 variants at the cross road of cajal body neuropathogenic mechanisms in distal neuropathies and motor neuron diseases. Neurobiol Dis. 2023;183:106172. 10.1016/j.nbd.2023.106172 37257665

[32] Turner BM. Nucleosome signalling; an evolving concept. Biochim Biophys Acta. 2014;1839(8):623–626. doi: 10.1016/j.bbagrm.2014.01.001 24412235

[33] Becker PB, Workman JL. Nucleosome remodeling and epigenetics. Cold Spring Harb Perspect Biol. 2013;5(9):a017905. doi: 10.1101/cshperspect.a017905 24003213 PMC3753709

[34] Monte-Serrano E, Morejón-García P, Campillo-Marcos I, et al. The pattern of histone H3 epigenetic posttranslational modifications is regulated by the VRK1 chromatin kinase. Epigenet Chromatin. 2023;16(1):18. doi: 10.1186/s13072-023-00494-7 PMC10182654 37179361

[35] Serafim RAM, de Souza Gama FH, Dutra LA, et al. Development of pyridine-based inhibitors for the human vaccinia-related kinases 1 and 2. ACS Med Chem Lett. 2019;10(9):1266–1271. doi: 10.1021/acsmedchemlett.9b00082 31531195 PMC6746079

[36] Vaquero A, Scher MB, Lee DH, et al. SirT2 is a histone deacetylase with preference for histone H4 lys 16 during mitosis. Genes Dev. 2006;20(10):1256–1261. doi: 10.1101/gad.1412706 16648462 PMC1472900

[37] Vaquero A, Sternglanz R, Reinberg D. Nad±dependent deacetylation of H4 lysine 16 by class III HDACs. Oncogene. 2007;26(37):5505–5520. doi: 10.1038/sj.onc.1210617 17694090

[38] Chakraborty S, Singh M, Pandita RK, Singh V, Lo CSC, Leonard F, Horikoshi N, Moros EG, Guha D, Hunt CR, et al. Heat-induced SIRT1-mediated H4K16ac deacetylation impairs resection and SMARCAD1 recruitment to double strand breaks. iScience. 2022;25(4):104142. doi: 10.1016/j.isci.2022.104142 35434547 PMC9010620

[39] Navarro-Carrasco E, Lazo PA. VRK1 depletion facilitates the synthetic lethality of temozolomide and olaparib in glioblastoma cells. Front Cell Dev Biol. 2021;9:683038. doi: 10.3389/fcell.2021.683038 34195200 PMC8237761

[40] Campillo-Marcos I, Monte-Serrano E, Navarro-Carrasco E, et al. Lysine methyltransferase inhibitors impair H4K20me2 and 53BP1 foci in response to DNA damage in sarcomas, a synthetic lethality strategy. Front Cell Dev Biol. 2021;9:715126. doi: 10.3389/fcell.2021.715126 34540832 PMC8446283

[41] Santos CR, Rodriguez-Pinilla M, Vega FM, et al. VRK1 signaling pathway in the context of the proliferation phenotype in head and neck squamous cell carcinoma. Mol Cancer Res. 2006;4(3):177–185. doi: 10.1158/1541-7786.MCR-05-0212 16547155

[42] Valbuena A, López-Sánchez I, Lazo PA, Williams S. Human VRK1 is an early response gene and its loss causes a block in cell cycle progression. PLOS ONE. 2008;3(2):e1642. doi: 10.1371/journal.pone.0001642 18286197 PMC2241669

[43] Lopez-Borges S, Lazo PA. The human vaccinia-related kinase 1 (VRK1) phosphorylates threonine-18 within the mdm-2 binding site of the p53 tumour suppressor protein. Oncogene. 2000;19(32):3656–3664. doi: 10.1038/sj.onc.1203709 10951572

[44] Sevilla A, Santos CR, Barcia R, et al. C-Jun phosphorylation by the human vaccinia-related kinase 1 (VRK1) and its cooperation with the N-terminal kinase of c-Jun (JNK). Oncogene. 2004;23(55):8950–8958. doi: 10.1038/sj.onc.1208015 15378002

[45] Sevilla A, Santos CR, Vega FM, et al. Human vaccinia-related kinase 1 (VRK1) activates the ATF2 transcriptional activity by novel phosphorylation on thr-73 and ser-62 and cooperates with JNK. J Biol Chem. 2004;279(26):27458–27465. doi: 10.1074/jbc.M401009200 15105425

[46] Kang TH, Park DY, Kim W, Kim KT. VRK1 phosphorylates CREB and mediates CCND1 expression. J Cell Sci. 2008;121(Pt 18):3035–3041. doi: 10.1242/jcs.026757 18713830

[47] Barcia R, Lopez-Borges S, Vega FM, et al. Kinetic properties of p53 phosphorylation by the human vaccinia-related kinase 1. Arch Biochem Biophys. 2002;399(1):1–5. doi: 10.1006/abbi.2001.2746 11883897

[48] Valbuena A, Suarez-Gauthier A, Lopez-Rios F, et al. Alteration of the VRK1-p53 autoregulatory loop in human lung carcinomas. Lung Cancer. 2007;58(3):303–309. doi: 10.1016/j.lungcan.2007.06.023 17689819

[49] Vega FM, Gonzalo P, Gaspar ML, et al. Expression of the VRK (vaccinia-related kinase) gene family of p53 regulators in murine hematopoietic development. FEBS Lett. 2003;544(1–3):176–180. doi: 10.1016/S0014-5793(03)00501-5 12782311

[50] Liu ZC, Cao K, Xiao ZH, et al. VRK1 promotes cisplatin resistance by up-regulating c-MYC via c-Jun activation and serves as a therapeutic target in esophageal squamous cell carcinoma. Oncotarget. 2017;8(39):65642–65658. doi: 10.18632/oncotarget.20020 29029460 PMC5630360

[51] Colmenero-Repiso A, Gómez-Muñoz MA, Rodríguez-Prieto I, et al. Identification of VRK1 as a new neuroblastoma tumor progression marker regulating cell proliferation. Cancers (Basel). 2020;12(11):3465. doi: 10.3390/cancers12113465 33233777 PMC7699843

[52] Sun X, Zhao W, Wang Q, Zhao J, Yang D, Yang Y. Inhibition of VRK1 suppresses proliferation and migration of vascular smooth muscle cells and intima hyperplasia after injury via mTorc1/β-catenin axis. BMB Rep. 2022;55(5):244–249. doi: 10.5483/BMBRep.2022.55.5.019 35410639 PMC9152580

[53] Carrión-Marchante R, Frezza V, Salgado-Figueroa A, et al. DNA aptamers against vaccinia-related kinase (VRK) 1 block proliferation in MCF7 breast cancer cells. Pharmaceuticals (Basel). 2021;14(5). 473. doi: 10.3390/ph14050473 34067799 PMC8156982

[54] Fernandez IF, Blanco S, Lozano J, et al. VRK2 inhibits mitogen-activated protein kinase signaling and inversely correlates with ErbB2 in human breast cancer. Mol Cell Biol. 2010;30(19):4687–4697. doi: 10.1128/MCB.01581-09 20679487 PMC2950518

[55] Fernandez IF, Perez-Rivas LG, Blanco S, Castillo-Dominguez AA, Lozano J, Lazo PA. VRK2 anchors KSR1-MEK1 to endoplasmic reticulum forming a macromolecular complex that compartmentalizes MAPK signaling. Cell Mol Life Sci. 2012;69(22):3881–3893. doi: 10.1007/s00018-012-1056-8 22752157 PMC11114894

[56] Vega FM, Sevilla A, Lazo PA. Lazo PA: p53 stabilization and accumulation induced by human vaccinia-related kinase 1. Mol Cell Biol. 2004;24(23):10366–10380. doi: 10.1128/MCB.24.23.10366-10380.2004 15542844 PMC529057

[57] Cartwright TN, Harris RJ, Meyer SK, Mon AM, Watson NA, Tan C, Marcelot A, Wang F, Zinn-Justin S, Traktman P, Higgins JMG. Dissecting the roles of haspin and VRK1 in histone H3 phosphorylation during mitosis. Sci Rep. 2022;12(1):11210. doi: 10.1038/s41598-022-15339-8 35778595 PMC9249732

[58] Ryu HG, Jung Y, Lee N, et al. HNRNP A1 promotes lung cancer cell proliferation by modulating VRK1 translation. Int J Mol Sci. 2021;22(11):5506. doi: 10.3390/ijms22115506 34071140 PMC8197126

[59] Choi YH, Lim JK, Jeong MW, et al. HnRNP A1 phosphorylated by VRK1 stimulates telomerase and its binding to telomeric DNA sequence. Nucleic Acids Res. 2012;40(17):8499–8518. doi: 10.1093/nar/gks634 22740652 PMC3458570

[60] Choi YH, Park CH, Kim W, Ling H, Kang A, Chang MW, Im SK, Jeong HW, Kong YY, Kim KT, Milstone DS. Vaccinia-related kinase 1 is required for the maintenance of undifferentiated spermatogonia in mouse male germ cells. PLOS ONE. 2010;5(12):e15254. doi: 10.1371/journal.pone.0015254 21179456 PMC3001494

[61] Wiebe MS, Nichols RJ, Molitor TP, et al. Mice deficient in the serine/threonine protein kinase VRK1 are infertile due to a progressive loss of spermatogonia. Biol Reprod. 2010;82(1):182–193. doi: 10.1095/biolreprod.109.079095 19696012 PMC2802121

[62] Schober CS, Aydiner F, Booth CJ, et al. The kinase VRK1 is required for normal meiotic progression in mammalian oogenesis. Mech Dev. 2011;128(3–4):178–190. doi: 10.1016/j.mod.2011.01.004 21277975

[63] Kelly AE, Ghenoiu C, Xue JZ, et al. Survivin reads phosphorylated histone H3 threonine 3 to activate the mitotic kinase aurora B. Science. 2010;330(6001):235–239. doi: 10.1126/science.1189505 20705815 PMC3177562

[64] Jeong MW, Kang TH, Kim W, et al. Mitogen-activated protein kinase phosphatase 2 regulates histone H3 phosphorylation via interaction with vaccinia-related kinase 1. Mol Biol Cell. 2013;24(3):373–384. doi: 10.1091/mbc.E12-06-0456 23223570 PMC3564537

[65] Yamagishi Y, Honda T, Tanno Y, et al. Two histone marks establish the inner centromere and chromosome bi-orientation. Science. 2010;330(6001):239–243. doi: 10.1126/science.1194498 20929775

[66] Berenguer I, López-Jiménez P, Mena I, Viera A, Page J, González-Martínez J, Maestre C, Malumbres M, Suja JA, Gómez R. Haspin participates in AURKB recruitment to centromeres and contributes to chromosome congression in male mouse meiosis. J Cell Sci. 2022;135(13). doi: 10.1242/jcs.259546 35694956

[67] Hirota T, Lipp JJ, Toh BH, et al. Histone H3 serine 10 phosphorylation by Aurora B causes HP1 dissociation from heterochromatin. Nature. 2005;438(7071):1176–1180. doi: 10.1038/nature04254 16222244

[68] Ivanovska I, Khandan T, Ito T, et al. A histone code in meiosis: the histone kinase, NHK-1, is required for proper chromosomal architecture in drosophila oocytes. Genes Dev. 2005;19(21):2571–2582. doi: 10.1101/gad.1348905 16230526 PMC1276731

[69] Lancaster OM, Breuer M, Cullen CF, et al. The meiotic recombination checkpoint suppresses NHK-1 kinase to prevent reorganisation of the oocyte nucleus in Drosophila. PLOS Genet. 2010;6(10):e1001179. doi: 10.1371/journal.pgen.1001179 21060809 PMC2965759

[70] Kim J, Choi YH, Chang S, et al. Defective folliculogenesis in female mice lacking vaccinia-related kinase 1. Sci Rep. 2012;2(1):468. doi: 10.1038/srep00468 22741057 PMC3384087

[71] Lancaster OM, Cullen CF, Ohkura H. NHK-1 phosphorylates BAF to allow karyosome formation in the drosophila oocyte nucleus. J Cell Bio. 2007;179(5):817–824. doi: 10.1083/jcb.200706067 18039935 PMC2099182

[72] Feinstein TN, Linstedt AD, Glick B. Mitogen-activated protein kinase kinase 1-dependent golgi unlinking occurs in G2 phase and promotes the G2/M cell cycle transition. Mol Biol Cell. 2007;18(2):594–604. doi: 10.1091/mbc.E06-06-0530 17182854 PMC1783781

[73] Shaul YD, Seger R. ERK1c regulates golgi fragmentation during mitosis. J Cell Bio. 2006;172(6):885–897. doi: 10.1083/jcb.200509063 16533948 PMC2063732

[74] Ruan Q, Wang Q, Xie S, et al. Polo-like kinase 3 is Golgi localized and involved in regulating Golgi fragmentation during the cell cycle. Exp Cell Res. 2004;294(1):51–59. doi: 10.1016/j.yexcr.2003.10.022 14980500

[75] Lopez-Sanchez I, Sanz-Garcia M, Lazo PA. Plk3 interacts with and specifically phosphorylates VRK1 in Ser342, a downstream target in a pathway that induces golgi fragmentation. Mol Cell Biol. 2009;29(5):1189–1201. doi: 10.1128/MCB.01341-08 19103756 PMC2643820

[76] Blanco S, Klimcakova L, Vega FM, et al. The subcellular localization of vaccinia-related kinase-2 (VRK2) isoforms determines their different effect on p53 stability in tumour cell lines. FEBS J. 2006;273(11):2487–2504. doi: 10.1111/j.1742-4658.2006.05256.x 16704422

[77] Sanz-Garcia M, Lopez-Sanchez I, Lazo PA. Proteomics identification of nuclear ran GTPase as an inhibitor of human VRK1 and VRK2 (vaccinia-related kinase) activities. Mol & Cell Proteomics. 2008;7(11):2199–2214. doi: 10.1074/mcp.M700586-MCP200 PMC2577208 18617507

[78] Adam S, Dabin J, Polo SE. Chromatin plasticity in response to DNA damage: the shape of things to come. DNA Repair. 2015;32:120–126. doi: 10.1016/j.dnarep.2015.04.022 25957486 PMC5111726

[79] Santos Á D, Cook AW, Gough RE, Schilling M, Olszok NA, Brown I, Wang L, Aaron J, Martin-Fernandez ML, Rehfeldt F, Toseland CP. DNA damage alters nuclear mechanics through chromatin reorganization. Nucleic Acids Res. 2021;49(1):340–353. doi: 10.1093/nar/gkaa1202 33330932 PMC7797048

[80] Navarro-Carrasco E, Campos-Díaz A, Monte-Serrano E, Rolfs F, de Goeij-de Haas R, Pham TV, Piersma SR, Jiménez CR, Lazo PA. Loss of VRK1 alters the nuclear phosphoproteome in the DNA damage response to doxorubicin. Chem Biol Interact. 2024;2024:110908. doi: 10.1016/j.cbi.2024.110908 38367682

[81] Navarro-Carrasco E, Monte-Serrano E, Campos-Díaz A, et al. VRK1 regulates sensitivity to oxidative stress by altering histone epigenetic modifications and the nuclear phosphoproteome in tumor cells. Int J Mol Sci. 2024;25(9):4874. doi: 10.3390/ijms25094874 38732093 PMC11084957

[82] Sanz-Garcia M, Monsalve DM, Sevilla A, et al. Vaccinia-related kinase 1 (VRK1) is an upstream nucleosomal kinase required for the assembly of 53BP1 foci in response to ionizing radiation-induced DNA damage. J Biol Chem. 2012;287(28):23757–23768. doi: 10.1074/jbc.M112.353102 22621922 PMC3390650

[83] Barcia-Sanjurjo I, Vazquez-Cedeira M, Barcia R, et al. Sensitivity of the kinase activity of human vaccinia-related kinase proteins to toxic metals. J Biol Inorg Chem. 2013;18(4):473–482. doi: 10.1007/s00775-013-0992-6 23483238

[84] Salzano M, Vazquez-Cedeira M, Sanz-Garcia M, Valbuena A, Blanco S, Fernandez IF, Lazo PA. Vaccinia-related kinase 1 (VRK1) confers resistance to DNA-damaging agents in human breast cancer by affecting DNA damage response. Oncotarget. 2014;5(N7):1770–1778. doi: 10.18632/oncotarget.1678 24731990 PMC4039124

[85] Monsalve DM, Campillo-Marcos I, Salzano M, Sanz-Garcia M, Cantarero L, Lazo PA. VRK1 phosphorylates and protects NBS1 from ubiquitination and proteasomal degradation in response to DNA damage. Biochim Biophys Acta Mol Cell Res. 2016;1863(4):760–769. doi: 10.1016/j.bbamcr.2016.02.005 26869104

[86] Martin-Doncel E, Rojas AM, Cantarero L, Lazo PA. VRK1 functional insufficiency due to alterations in protein stability or kinase activity of human VRK1 pathogenic variants implicated in neuromotor syndromes. Sci Rep. 2019;9(1):13381. doi: 10.1038/s41598-019-49821-7 31527692 PMC6746721

[87] Navarro-Carrasco E, Campos-Díaz A, Monte-Serrano E, Rolfs F, de Goeij-de Haas R, Pham TV, Piersma SR, Jiménez CR, Lazo PA. Loss of VRK1 alters the nuclear phosphoproteome in the DNA damage response to doxorubicin. Chem Biol Interact. 2024;391:110908. doi: 10.1016/j.cbi.2024.110908 38367682

[88] Campillo-Marcos I, Lazo PA. Olaparib and ionizing radiation trigger a cooperative DNA-damage repair response that is impaired by depletion of the VRK1 chromatin kinase. J Exp Clin Cancer Res. 2019;38(1):203. doi: 10.1186/s13046-019-1204-1 31101118 PMC6525392

[89] Maynard S, Fang EF, Scheibye-Knudsen M, et al. DNA damage, DNA repair, aging, and neurodegeneration. Cold Spring Harb Perspect Med. 2015;5(10):a025130. doi: 10.1101/cshperspect.a025130 26385091 PMC4588127

[90] Sun Y, Curle AJ, Haider AM, Balmus G. The role of DNA damage response in amyotrophic lateral sclerosis. Essays Biochem. 2020;64(5):847–861. doi: 10.1042/ebc20200002 33078197 PMC7588667

[91] Madabhushi R, Pan L, Tsai LH. DNA damage and its links to neurodegeneration. Neuron. 2014;83(2):266–282. doi: 10.1016/j.neuron.2014.06.034 25033177 PMC5564444

[92] Lafarga M, Tapia O, Romero AM, et al. Cajal bodies in neurons. RNA Biol. 2017;14(6):712–725. doi: 10.1080/15476286.2016.1231360 27627892 PMC5519235

[93] Tapia O, Narcís JO, Riancho J, et al. Cellular bases of the RNA metabolism dysfunction in motor neurons of a murine model of spinal muscular atrophy: role of cajal bodies and the nucleolus. Neurobiol Dis. 2017;108:83–99. doi: 10.1016/j.nbd.2017.08.004 28823932

[94] Singh RN, Howell MD, Ottesen EW, et al. Diverse role of survival motor neuron protein. Biochim Biophys Acta Gene Regul Mech. Biochim Biophys Acta Gene Regul Mech. 2017;1860(3):299–315. doi: 10.1016/j.bbagrm.2016.12.008 28095296 PMC5325804

[95] Biton S, Barzilai A, Shiloh Y. The neurological phenotype of ataxia-telangiectasia: solving a persistent puzzle. DNA Repair. 2008;7(7):1028–1038. doi: 10.1016/j.dnarep.2008.03.006 18456574

[96] Lavin MF. ATM and the Mre11 complex combine to recognize and signal DNA double-strand breaks. Oncogene. 2007;26(56):7749–7758. doi: 10.1038/sj.onc.1210880 18066087

[97] Houldsworth A. Role of oxidative stress in neurodegenerative disorders: a review of reactive oxygen species and prevention by antioxidants. Brain Com. 2024;6(1):fcad356. doi: 10.1093/braincomms/fcad356 PMC10783645 38214013

[98] Toledo F, Wahl GM. Regulating the p53 pathway: in vitro hypotheses, in vivo veritas. Nat Rev Cancer. 2006;6(12):909–923. doi: 10.1038/nrc2012 17128209

[99] Meek DW. Tumour suppression by p53: a role for the DNA damage response? Nat Rev Cancer. 2009;9(10):714–723. doi: 10.1038/nrc2716 19730431

[100] Lazo PA. Reverting p53 activation after recovery of cellular stress to resume with cell cycle progression. Cell Signal. 2017;33:49–58. doi: 10.1016/j.cellsig.2017.02.005 28189587

[101] Lopez-Sanchez I, Valbuena A, Vazquez-Cedeira M, et al. VRK1 interacts with p53 forming a basal complex that is activated by UV-induced DNA damage. FEBS Lett. 2014;588(5):692–700. doi: 10.1016/j.febslet.2014.01.040 24492002

[102] Valbuena A, Vega FM, Blanco S. Lazo PA: p53 downregulates its activating vaccinia-related kinase 1, forming a new autoregulatory loop. Mol Cell Biol. 2006;26(13):4782–4793. doi: 10.1128/MCB.00069-06 16782868 PMC1489172

[103] Schon O, Friedler A, Bycroft M, et al. Molecular mechanism of the interaction between MDM2 and p53. J Mol Biol. 2002;323(3):491–501. doi: 10.1016/S0022-2836(02)00852-5 12381304

[104] Teufel DP, Bycroft M, Fersht AR. Regulation by phosphorylation of the relative affinities of the N-terminal transactivation domains of p53 for p300 domains and Mdm2. Oncogene. 2009;28(20):2112–2118. doi: 10.1038/onc.2009.71 19363523 PMC2685776

[105] Oren M. Decision making by p53: life, death and cancer. Cell Death Differ. 2003;10(4):431–442. doi: 10.1038/sj.cdd.4401183 12719720

[106] Valbuena A, Castro-Obregon S, Lazo PA, Wu GS. Downregulation of VRK1 by p53 in response to DNA Damage is Mediated by the autophagic pathway. PLOS ONE. 2011;6(2):e17320. doi: 10.1371/journal.pone.0017320 21386980 PMC3046209

[107] Valbuena A, Blanco S, Vega FM, Lazo PA, Fugmann SD. The C/H3 domain of p300 is required to protect VRK1 and VRK2 from their downregulation induced by p53. PLOS ONE. 2008;3(7):e2649. doi: 10.1371/journal.pone.0002649 18612383 PMC2441436

[108] Gorjanacz M, Klerkx EP, Galy V, et al. Caenorhabditis elegans BAF-1 and its kinase VRK-1 participate directly in post-mitotic nuclear envelope assembly. Embo J. 2008;26(1):132–143. doi: 10.1038/sj.emboj.7601470 PMC1782363 17170708

[109] Burger M, Schmitt-Koopmann C, Leroux JC. DNA unchained: two assays to discover and study inhibitors of the DNA clustering function of barrier-to-autointegration factor. Sci Rep. 2020;10(1):12301. doi: 10.1038/s41598-020-69246-x 32704141 PMC7378220

[110] Zheng R, Ghirlando R, Lee MS, et al. Barrier-to-autointegration factor (BAF) bridges DNA in a discrete, higher-order nucleoprotein complex. Proc Natl Acad Sci USA. 2000;97(16):8997–9002. doi: 10.1073/pnas.150240197 10908652 PMC16810

[111] Margalit A, Segura-Totten M, Gruenbaum Y, et al. Barrier-to-autointegration factor is required to segregate and enclose chromosomes within the nuclear envelope and assemble the nuclear lamina. Proc Natl Acad Sci USA. 2005;102(9):3290–3295. doi: 10.1073/pnas.0408364102 15728376 PMC552915

[112] Montes de Oca R, Shoemaker CJ, Gucek M, et al. Barrier-to-autointegration factor proteome reveals chromatin-regulatory partners. PLOS ONE. 2009;4(9):e7050. doi: 10.1371/journal.pone.0007050 19759913 PMC2739719

[113] Nichols RJ, Wiebe MS, Traktman P. The vaccinia-related kinases phosphorylate the N’ terminus of BAF, regulating its interaction with DNA and its retention in the nucleus. Mol Biol Cell. 2006;17(5):2451–2464. doi: 10.1091/mbc.E05-12-1179 16495336 PMC1446082

[114] Molitor TP, Traktman P, Hetzer M. Depletion of the protein kinase VRK1 disrupts nuclear envelope morphology and leads to BAF retention on mitotic chromosomes. Mol Biol Cell. 2014;25(6):891–903. doi: 10.1091/mbc.E13-10-0603 24430874 PMC3952857

[115] Torras-Llort M, Medina-Giró S, Escudero-Ferruz P, et al. A fraction of barrier-to-autointegration factor (BAF) associates with centromeres and controls mitosis progression. Commun Biol. 2020;3(1):454. doi: 10.1038/s42003-020-01182-y 32814801 PMC7438335

[116] Jamin A, Wiebe MS. Barrier to autointegration factor (BANF1): interwoven roles in nuclear structure, genome integrity, innate immunity, stress responses and progeria. Curr Opin Cell Biol. 2015;34:61–68. doi: 10.1016/j.ceb.2015.05.006 26072104 PMC4522355

[117] Bengtsson L, Wilson KL. Barrier-to-autointegration factor phosphorylation on ser-4 regulates emerin binding to lamin a in vitro and emerin localization in vivo. Mol Biol Cell. 2006;17(3):1154–1163. doi: 10.1091/mbc.e05-04-0356 16371512 PMC1382305

[118] Skoko D, Li M, Huang Y, et al. Barrier-to-autointegration factor (BAF) condenses DNA by looping. Proc Natl Acad Sci USA. 2009;106(39):16610–16615. doi: 10.1073/pnas.0909077106 19805345 PMC2748091

[119] Zhuang X, Semenova E, Maric D, et al. Dephosphorylation of barrier-to-autointegration factor by protein phosphatase 4 and its role in cell mitosis. J Biol Chem. 2014;289(2):1119–1127. doi: 10.1074/jbc.M113.492777 24265311 PMC3887179

[120] Marcelot A, Petitalot A, Ropars V, et al. Di-phosphorylated BAF shows altered structural dynamics and binding to DNA, but interacts with its nuclear envelope partners. Nucleic Acids Res. 2021;49(7):3841–3855. doi: 10.1093/nar/gkab184 33744941 PMC8053085

[121] Ren Z, Geng J, Xiong C, et al. Downregulation of VRK1 reduces the expression of BANF1 and suppresses the proliferative and migratory activity of esophageal cancer cells. Oncol Lett. 2020;20(2):1163–1170. doi: 10.3892/ol.2020.11654 32724356 PMC7377186

[122] Asencio C, Davidson IF, Santarella-Mellwig R, et al. Coordination of kinase and phosphatase activities by Lem4 enables nuclear envelope reassembly during mitosis. Cell. 2012;150(1):122–135. doi: 10.1016/j.cell.2012.04.043 22770216

[123] Gorjanacz M. LEM-4 promotes rapid dephosphorylation of BAF during mitotic exit. Nucleus. 2012;4(1):14–17. doi: 10.4161/nucl.22961 23211644 PMC3585021

[124] Kaufmann T, Kukolj E, Brachner A, et al. SIRT2 regulates nuclear envelope reassembly through ANKLE2 deacetylation. J Cell Sci. 2016;129(24):4607–4621. doi: 10.1242/jcs.192633 27875273 PMC5201015

[125] Apridita Sebastian W, Shiraishi H, Shimizu N, et al. Ankle2 deficiency-associated microcephaly and spermatogenesis defects in zebrafish are alleviated by heterozygous deletion of vrk1. Biochem Biophys Res Commun. 2022;624:95–101. doi: 10.1016/j.bbrc.2022.07.070 35940133

[126] Carrasco Apolinario ME, Umeda R, Teranishi H, Shan M, Phurpa SW, Lai S, Shimizu N, Shiraishi H, Shikano K, et al. Behavioral and neurological effects of Vrk1 deficiency in zebrafish. Biochem Biophys Res Commun. 2023;675:10–18. doi: 10.1016/j.bbrc.2023.07.005 37429068

[127] Gonzaga-Jauregui C, Lotze T, Jamal L, et al. Mutations in VRK1 associated with complex motor and sensory axonal neuropathy plus microcephaly. JAMA Neurol. 2013;70(12):1491–1498. doi: 10.1001/jamaneurol.2013.4598 24126608 PMC4039291

[128] Bradley CM, Ronning DR, Ghirlando R, et al. Structural basis for DNA bridging by barrier-to-autointegration factor. Nat Struct Mol Biol. 2005;12(10):935–936. doi: 10.1038/nsmb989 16155580

[129] Burgess JT, Cheong CM, Suraweera A, et al. Barrier-to-autointegration-factor (Banf1) modulates DNA double-strand break repair pathway choice via regulation of DNA-dependent kinase (DNA-PK) activity. Nucleic Acids Res. 2021;49(6):3294–3307. doi: 10.1093/nar/gkab110 33660778 PMC8034644

[130] Bolderson E, Burgess JT, Li J, Gandhi NS, Boucher D, Croft LV, Beard S, Plowman JJ, Suraweera A, Adams MN, et al. Barrier-to-autointegration factor 1 (Banf1) regulates poly [ADP-ribose] polymerase 1 (PARP1) activity following oxidative DNA damage. Nat Commun. 2019;10(1):5501. doi: 10.1038/s41467-019-13167-5 31796734 PMC6890647

[131] Prissette M, Fury W, Koss M, et al. Disruption of nuclear envelope integrity as a possible initiating event in tauopathies. Cell Rep. 2022;40(8):111249. doi: 10.1016/j.celrep.2022.111249 36001963

[132] Ramon y Cajal S. Un sencillo metodo de coloracion selectiva del reticulo protoplasmatico y sus efectos en los diversos organos nerviosos de vertebrados e invertebrados. Trab Lab Invest Biol (Madrid). 1903;2:129–221.

[133] Hebert MD, Matera AG, Silver PA. Self-association of coilin reveals a common theme in nuclear body localization. Mol Biol Cell. 2000;11(12):4159–4171. doi: 10.1091/mbc.11.12.4159 11102515 PMC15064

[134] Hebert MD, Poole AR. Towards an understanding of regulating cajal body activity by protein modification. RNA Biol. 2017;14(6):761–778. doi: 10.1080/15476286.2016.1243649 27819531 PMC5519237

[135] Staněk D. Coilin and Cajal bodies. Nucleus. 2023;14(1):2256036. doi: 10.1080/19491034.2023.2256036 37682044 PMC10494742

[136] Machyna M, Heyn P, Neugebauer KM. Cajal bodies: where form meets function. Wiley Interdiscip Rev RNA. 2013;4(1):17–34. doi: 10.1002/wrna.1139 23042601

[137] Hearst SM, Gilder AS, Negi SS, Davis MD, George EM, Whittom AA, Toyota CG, Husedzinovic A, Gruss OJ, Hebert MD. Cajal-body formation correlates with differential coilin phosphorylation in primary and transformed cell lines. J Cell Sci. 2009;122(Pt 11):1872–1881 doi: 10.1242/jcs.044040 19435804 PMC2684838

[138] Geisler MS, Kemp JP Jr., Duronio RJ. Duronio RJ: histone locus bodies: a paradigm for how nuclear biomolecular condensates control cell cycle regulated gene expression. Nucleus. 2023;14(1):2293604. doi: 10.1080/19491034.2023.2293604 38095604 PMC10730174

[139] Suzuki H, Abe R, Shimada M, et al. The 3’ pol II pausing at replication-dependent histone genes is regulated by mediator through cajal bodies’ association with histone locus bodies. Nat Commun. 2022;13(1):2905. doi: 10.1038/s41467-022-30632-w 35614107 PMC9133132

[140] Arias Escayola D, Neugebauer KM. Dynamics and function of nuclear bodies during embryogenesis. Biochemistry. 2018;57(17):2462–2469. doi: 10.1021/acs.biochem.7b01262 29473743

[141] Nizami Z, Deryusheva S, Gall JG. The cajal body and histone locus body. Cold Spring Harbor Perspect Biol. 2010;2(7):a000653. doi: 10.1101/cshperspect.a000653 PMC2890199 20504965

[142] Machyna M, Kehr S, Straube K, et al. The coilin interactome identifies hundreds of small noncoding RNAs that traffic through cajal bodies. Mol Cell. 2014;56(3):389–399. doi: 10.1016/j.molcel.2014.10.004 25514182

[143] Morejon-Garcia P, Keren B, Marcos-Alcalde I, et al. Dysfunctional homozygous VRK1-D263G variant impairs the Assembly of Cajal Bodies and DNA damage response in hereditary spastic paraplegia. Neurol Genet. 2021;7(5):e624. doi: 10.1212/nxg.0000000000000624 34504951 PMC8422991

[144] Marcos AT, Martin-Doncel E, Morejon-Garcia P, et al. VRK1 (Y213H) homozygous mutant impairs cajal bodies in a hereditary case of distal motor neuropathy. Ann Clin Transl Neurol. 2020;7(5):808–818. doi: 10.1002/acn3.51050 32365420 PMC7261760

[145] Bos R, Rihan K, Quintana P, El-Bazzal L, Bernard-Marissal N, Da Silva N, Jabbour R, Mégarbané A, Bartoli M, Brocard F, Delague V. Altered action potential waveform and shorter axonal initial segment in hiPSC-derived motor neurons with mutations in VRK1. Neurobiology of Disease. 2022;2022:105609. doi: 10.1016/j.nbd.2021.105609 34990802

[146] Lee YJ, Rio DC. A mutation in the low-complexity domain of splicing factor hnRNPA1 linked to amyotrophic lateral sclerosis disrupts distinct neuronal RNA splicing networks. Genes Dev. 2024;38(1–2):11–30. doi: 10.1101/gad.351104.123 38182429 PMC10903937

[147] Beijer D, Kim HJ, Guo L, et al. Characterization of HNRNPA1 mutations defines diversity in pathogenic mechanisms and clinical presentation. JCI Insight. 2021;6(14). doi: 10.1172/jci.insight.148363 PMC8410042 34291734

[148] Molitor TP, Traktman P. Molecular genetic analysis of VRK1 in mammary epithelial cells: depletion slows proliferation in vitro and tumor growth and metastasis in vivo. Oncogenesis. 2013;2(6):e48. doi: 10.1038/oncsis.2013.11 23732708 PMC3740298

[149] Mon AM, MacKinnon AC Jr., Traktman P. Traktman P: overexpression of the VRK1 kinase, which is associated with breast cancer, induces a mesenchymal to epithelial transition in mammary epithelial cells. PLOS ONE. 2018;13(9):e0203397. doi: 10.1371/journal.pone.0203397 30180179 PMC6122820

[150] Liu J, Wang Y, He S, et al. Expression of vaccinia-related kinase 1 (VRK1) accelerates cell proliferation but overcomes cell adhesion mediated drug resistance (CAM-DR) in multiple myeloma. Hematology. 2016;21(10):603–612. doi: 10.1080/10245332.2016.1147678 27319807 PMC9491125

[151] Du N, Zhang B, Zhang Y. Downregulation of VRK1 inhibits progression of lung squamous cell carcinoma through DNA damage. Can Respir J. 2023;2023:4533504. doi: 10.1155/2023/4533504 37547297 PMC10403328

[152] Li J, Wang T, Pei L, et al. Expression of VRK1 and the downstream gene BANF1 in esophageal cancer. Biomed Pharmacother. 2017;89:1086–1091. doi: 10.1016/j.biopha.2017.02.095 28298069

[153] Wang L, Zhai R, Shen H, et al. VRK1 promotes proliferation, migration, and invasion of gastric carcinoma cells by activating β-catenin. Neoplasma. 2021;68(5):1005–1014. doi: 10.4149/neo_2021_210304N278 34374295

[154] Chen D, Zhou W, Chen J, Wang J. Comprehensively prognostic and immunological analysis of VRK Serine/Threonine kinase 1 in pan-cancer and identification in hepatocellular carcinoma. Aging (Albany NY). 2023;15(24):15504–15524. doi: 10.18632/aging.205389 38157278 PMC10781469

[155] Hennig EE, Mikula M, Rubel T, Dadlez M, Ostrowski J. Comparative kinome analysis to identify putative colon tumor biomarkers. J Mol Med (Berl). 2012;90(4):447–456. doi: 10.1007/s00109-011-0831-6 22095101 PMC3307991

[156] Martin KJ, Patrick DR, Bissell MJ, Fournier MV, Preiss T. Prognostic breast cancer signature identified from 3D culture model accurately predicts clinical outcome across independent datasets. PLOS ONE. 2008;3(8):e2994. doi: 10.1371/journal.pone.0002994 18714348 PMC2500166

[157] Zhao X, Chu X, Song L, et al. A novel model incorporating chromatin regulatory factors for risk stratification, prognosis prediction, and characterization of the microenvironment in Wilms tumor. The Journal of Gene Medicine. 2024;2023(1):e3574. doi: 10.1002/jgm.3574 37578081

[158] Ben Z, Gong L, Qiu Y. High expression of VRK1 is related to poor prognosis in glioma. Pathol Res Pract. 2018;214(1):112–118. doi: 10.1016/j.prp.2017.10.014 29103766

[159] So J, Mabe NW, Englinger B, Chow KH, Moyer SM, Yerrum S, Trissal MC, Marques JG, Kwon JJ, Shim B, et al. VRK1 as a synthetic lethal target in VRK2 promoter-methylated cancers of the nervous system. JCI Insight. 2022;7(19). doi: 10.1172/jci.insight.158755 PMC9675470 36040810

[160] Varghese RT, Liang Y, Guan T, et al. Survival kinase genes present prognostic significance in glioblastoma. Oncotarget. 2016;7(15):20140–20151. doi: 10.18632/oncotarget.7917 26956052 PMC4991443

[161] Eswaran J, Patnaik D, Filippakopoulos P, Wang F, Stein RL, Murray JW, Higgins JM, Knapp S. Structure and functional characterization of the atypical human kinase haspin. Proc Natl Acad Sci USA. 2009;106(48):20198–20203. doi: 10.1073/pnas.0901989106 19918057 PMC2777956

[162] Fedorov O, Marsden B, Pogacic V, et al. A systematic interaction map of validated kinase inhibitors with ser/thr kinases. Proc Natl Acad Sci USA. 2007;104(51):20523–20528. doi: 10.1073/pnas.0708800104 18077363 PMC2154464

[163] Fedorov O, Sundstrom M, Marsden B, et al. Insights for the development of specific kinase inhibitors by targeted structural genomics. Drug Discov Today. 2007;12(9–10):365–372. doi: 10.1016/j.drudis.2007.03.006 17467572

[164] Vazquez-Cedeira M, Barcia-Sanjurjo I, Sanz-Garcia M, et al. Differential inhibitor sensitivity between human kinases VRK1 and VRK2. PLOS ONE. 2011;6(8):e23235. doi: 10.1371/journal.pone.0023235 21829721 PMC3150407

[165] Ngow YS, Rajan S, Ye H, Yoon HS. Crystal structure of human Vaccinia-related kinase 1 (VRK1) in complex with AMP-PNP, a non-hydrolysable ATP analog. Protein Science. 2019;28(3):524–532. doi: 10.1002/pro.3552 30461091 PMC6371211

[166] Couñago RM, Allerston CK, Savitsky P, et al. Structural characterization of human vaccinia-related kinases (VRK) bound to small-molecule inhibitors identifies different P-loop conformations. Sci Rep. 2017;7(1):7501. doi: 10.1038/s41598-017-07755-y 28790404 PMC5548783

[167] Dawson MA, Kouzarides T. Cancer epigenetics: from mechanism to therapy. Cell. 2012;150(1):12–27. doi: 10.1016/j.cell.2012.06.013 22770212

[168] Simó-Riudalbas L, Esteller M. Targeting the histone orthography of cancer: drugs for writers, erasers and readers. Br J Pharmacol. 2015;172(11):2716–2732. doi: 10.1111/bph.12844 25039449 PMC4439870

[169] Fradet-Turcotte A, Canny MD, Escribano-Díaz C, Orthwein A, Leung CC, Huang H, Landry MC, Kitevski-LeBlanc J, Noordermeer SM, Sicheri F, Durocher D. 53BP1 is a reader of the DNA-damage-induced H2A lys 15 ubiquitin mark. Nature. 2013;499(7456):50–54. doi: 10.1038/nature12318 23760478 PMC3955401

[170] Tang J, Cho NW, Cui G, et al. Acetylation limits 53BP1 association with damaged chromatin to promote homologous recombination. Nat Struct Mol Biol. 2013;20(3):317–325. doi: 10.1038/nsmb.2499 23377543 PMC3594358

[171] He X, Zai G, Zhou L, et al. Identification of VRK1 as a novel potential biomarker for prognosis and immunotherapy in hepatocellular carcinoma. J Inflamm Res. 2024;17:1671–1683. doi: 10.2147/JIR.S452505 38504696 PMC10948335

[172] Fan Z, Wang X, Cheng H, Pan M. VRK1 promotes DNA-induced type I interferon production. Mol Biol Rep. 2024;51(1):453. doi: 10.1007/s11033-024-09414-8 38536553

[173] Shields JA, Meier SR, Bandi M, Mulkearns-Hubert EE, Hajdari N, Dam Ferdinez M, Engel JL, Silver DJ, Shen B, Zhang W, et al. VRK1 is a synthetic–lethal target in VRK2-deficient glioblastoma. Cancer Research. 2022. 82(21):4044–4057. doi: 10.1158/0008-5472.Can-21-4443 36069976 PMC9627132

